# Molecular Pharmacology at the Crossroads of Precision Medicine

**DOI:** 10.3390/cimb48010039

**Published:** 2025-12-27

**Authors:** Alina Crenguţa Nicolae, Ion-Bogdan Dumitrescu, Cristina Manuela Drăgoi

**Affiliations:** 1 Department of Biochemistry, Faculty of Pharmacy, Carol Davila University of Medicine and Pharmacy, RO-020956 Bucharest, Romania; alina.nicolae@umfcd.ro (A.C.N.); cristina.dragoi@umfcd.ro (C.M.D.); 2 Department of Physics and Informatics, Faculty of Pharmacy, Carol Davila University of Medicine and Pharmacy, RO-020956 Bucharest, Romania

## 1. Molecular Pharmacology at the Intersection of Fundamental Molecular Science and Innovative Drug Development

Molecular pharmacology sits at the nexus of fundamental molecular science and therapeutic innovation. This field has seen remarkable progress over recent years, driven by advances in our understanding of drug–target interactions, disease pathways, and human genetics. Landmark achievements in molecular pharmacology have reshaped drug development paradigms, perhaps most notably through the shift from empirical, one-size-fits-all treatments to mechanism-guided targeted therapies and precision medicine approaches [1,2,3,4,5,6,7,8]. For example, recent analyses indicate that the majority of novel drugs approved in recent years qualify as targeted therapies, underscoring a decisive move toward agents that specifically modulate defined molecular drivers of disease. In parallel, over one-third of new FDA approvals now fall under personalized medicine, treatments tailored to patients’ individual biomarker or genetic profiles [9,10,11,12,13,14]. These trends highlight a fundamental evolution: modern pharmacology strives for the “five rights” of therapy—the right patient, drug, dose, time, and route—by leveraging molecular insights [15]. This transformation has only been possible through decades of fundamental research that elucidated the complex biology of receptors, enzymes, and signaling pathways and revealed how genetic variations influence drug response. It has become increasingly clear that pharmacogenomic factors—an individual’s genetic makeup affecting drug metabolism and targets—are critical to optimizing efficacy and safety. Indeed, pharmacogenomics has emerged as a cornerstone of precision pharmacology, tailoring drug selection and dosing to patients’ genetic features [15]. International consortia have even begun issuing clinical guidelines for genotype-guided prescribing, though their adoption in practice is still ongoing. Taken together, these developments mark a new era in which molecular pharmacology is not only about understanding how drugs work, but also about who they work for and why [16,17,18,19,20,21,22,23].

## 2. Privileged Scaffolds and Rational Drug Design

Amid this paradigm shift, drug discovery strategies have been refined by incorporating medicinal chemistry innovations. One concept gaining renewed prominence is the use of privileged scaffolds—structural motifs known to engage a variety of biological targets with high affinity. These scaffolds often possess favorable drug-like properties (e.g., suitable size, polarity, and metabolic stability), making them attractive templates in designing new therapeutics [4,24,25,26,27,28,29]. By cleverly modifying such privileged frameworks, researchers can efficiently generate libraries of candidate compounds with a higher prior probability of biological activity. The payoff of this approach is evidenced by its success across multiple domains, perhaps most visibly in antiviral and anticancer research. Privileged structures display versatile binding properties and can provide potent, selective ligands for diverse targets, yielding high-quality lead compounds and accelerating the development of novel drugs [4]. A striking recent example comes from the battle against COVID-19: researchers synthesized thiazole–pyridine hybrid molecules—built on a known bioactive scaffold—and demonstrated their efficacy in inhibiting the SARS-CoV-2 main protease in preclinical assays [30,31,32,33]. Such molecules, designed with structural insight, represent promising antiviral leads and underscore how scaffold-based drug design can rapidly respond to emerging health threats. More generally, leveraging privileged scaffolds allows medicinal chemists to simultaneously explore multiple therapeutic areas, repurposing core chemical structures to address different diseases. This strategy, grounded in fundamental structure–activity knowledge, exemplifies how rational drug design is superseding brute-force screening: researchers now design molecules with target binding and pharmacokinetic properties in mind from the outset, rather than relying purely on trial-and-error [34,35,36,37,38].

## 3. New Approaches Expanding the Pharmacological Arsenal

Another landmark achievement in molecular pharmacology has been the advent of entirely new therapeutic modalities beyond traditional small-molecule inhibitors. One notable innovation is the development of PROTACs (proteolysis-targeting chimeras) and related targeted protein degradation technologies. Unlike conventional drugs, which simply inhibit protein function, PROTACs recruit unwanted proteins to the cell’s own degradation machinery, thereby selectively eliminating disease-driving proteins. These chimeras represent an advanced therapeutic strategy that enables selective protein degradation, opening new possibilities in drug design [39,40], especially toward tackling targets previously deemed “undruggable,” such as scaffolding or transcriptional proteins without obvious active sites. Early PROTAC successes in preclinical models of cancer, viral infection, and neurodegenerative disease hint at their vast potential [41,42], and the first PROTAC-based candidates have now entered clinical trials, validating targeted protein degradation as more than a theoretical concept [43,44]. In a broader sense, molecular pharmacology has embraced an expanding toolkit that includes monoclonal antibodies, recombinant proteins, gene and cell therapies, and mRNA-based therapeutics. Each of these modalities stems from fundamental discoveries in molecular biology—from understanding the immune checkpoint receptors, yielding antibody immunotherapies, to deciphering mRNA translation mechanisms, paving the way for mRNA vaccines. The integration of these new therapeutic classes has blurred the boundaries between traditional pharmacology and biotechnology, creating a more integrative “pharmaco-biology.” Yet, all these advances share a common thread: they exploit molecular-level understanding of disease processes. For instance, the success of mRNA vaccines for COVID-19 demonstrated how precisely delivering genetic instructions to cells can induce pharmacological effects with unprecedented speed and specificity. As we harness these new approaches, molecular pharmacologists are adapting principles of drug action to novel contexts—whether the “drug” is a small organic molecule, a biomacromolecule, or even a living cell, the goal remains to modulate molecular pathways in a predictable, beneficial manner [45,46,47,48].

## 4. Precision and Personalized Pharmacology

Perhaps the most transformative progress has come in aligning therapies with patient-specific molecular profiles—the era of precision medicine. Fundamental research into genomic and phenotypic heterogeneity has revealed why patients with the same diagnosis can respond very differently to a given drug, fueling efforts to identify biomarkers (genetic mutations, receptor expression levels, metabolic signatures, etc.) that predict drug response or toxicity. The result is a growing roster of tailored therapies guided by companion diagnostics: from EGFR inhibitors given only to lung cancer patients with EGFR mutations, to CFTR modulators effective in cystic fibrosis patients with specific CFTR gene variants. Such targeted therapies exemplify how molecular pharmacology translates basic insights into clinical benefit. As noted, a majority of new drug approvals are now biomarker-driven agents, a testament to how deeply personalization has permeated pharmacological research and development. Personalized medicine considers each patient’s unique genetic and molecular makeup, disease subtype, environment, and lifestyle to craft the optimal treatment strategy [15,49]. Beyond selecting the right drug, this includes individualizing the dose (pharmacogenetics has taught us, for example, how variants in drug-metabolizing enzymes like CYP450s demand dose adjustments) and even the timing of administration [18,50,51,52,53,54,55]. The benefits of this approach are manifold: higher efficacy, by targeting drugs to those most likely to benefit; fewer adverse effects, by avoiding treatment of those likely to be harmed or non-responsive; and often cost-effectiveness for healthcare systems in the long run. A vivid illustration of precision pharmacology in action is the dramatic improvement in outcomes for certain cancers—oncogene-targeted therapies have turned once-lethal malignancies into manageable conditions for many patients [56,57,58,59,60]. Likewise, the explosion of checkpoint inhibitor immunotherapies, while not “targeted” to a mutation per se, relies on molecular understanding of tumor immune evasion and often uses biomarkers (like PD-L1 expression or MSI status) to guide use. Crucially, precision pharmacology extends beyond oncology: from cardiology to psychiatry, researchers are identifying genetic markers and endotypes of disease that correlate with drug response [8,61,62,63,64,65]. These advances owe a great debt to fundamental research in molecular genetics, clinical pharmacology, and systems biology, which continues to unravel the sources of inter-individual variability in drug effects.

## 5. Natural Products and Novel Chemical Matter from Fundamental Insights

It is worth noting that even as cutting-edge technologies flourish, classical pharmacology—often inspired by nature’s chemical diversity—remains an invaluable font of new therapeutic leads. Many “privileged” natural product scaffolds are inherently bioactive and have co-evolved with biological systems, making them excellent starting points for drug discovery. Natural molecules such as alkaloids, flavonoids, terpenes, and peptides have long been medicines or prototypes for synthetic drugs [37,66,67,68,69,70,71,72]. Today, with advanced tools to analyze complex extracts and modify natural structures, researchers are revisiting natural sources for new pharmacophores [73]. A striking example is the realization that fruit peels and other agricultural by-products, often discarded in massive quantities, contain a wealth of bioactive compounds with medicinal potential. Recent investigations showed that, in many cases, fruit peels harbor higher concentrations of beneficial phytochemicals and pharmacologically active molecules than fruit pulp itself [74]. Orange peel extracts, for instance, are rich in polymethoxylated flavones with anti-inflammatory and cholesterol-lowering effects, and citrus-peel-derived compounds have even entered trials as anti-cancer and cardioprotective agents. Hussain et al. comprehensively reviewed fruit peel constituents and highlighted their antimicrobial, antioxidant, anti-diabetic, and neuroprotective activities, advocating the repurposing of “food waste” into a resource for drug discovery [74,75]. Such work exemplifies how fundamental phytochemical and pharmacological research can transform our therapeutic armamentarium while also promoting sustainability. Another area of renewed interest is the pharmacology of dietary compounds and nutraceuticals. For example, polyphenols found in foods and beverages are being studied for their complex effects on human health [76,77,78,79,80]. Resveratrol and catechins garnered attention for cardioprotective and anti-aging properties, and more recently, red wine polyphenols have undergone rigorous evaluation [81,82]. A comprehensive review cataloged the myriad bioactivities of red-wine-derived polyphenols—including anti-inflammatory, anti-carcinogenic, and cardioprotective effects—in models of cardiovascular disease, cancer, and metabolic and gut microbiome modulation [83,84]. However, as the authors and others acknowledge, these nutraceutical effects can be subtle and context-dependent; indeed, years of study have yielded controversy and no clear consensus on some claims, underscoring the need for further fundamental and clinical research to validate benefits. The enduring message is that natural product pharmacology continues to inspire modern drug discovery, and rigorous molecular investigations are critical to separating genuine therapeutic potential from myths. By applying cutting-edge analytical techniques and mechanistic assays, researchers are translating traditional knowledge and chemical biodiversity into new pharmacological opportunities, often discovering privileged structures in the process [85,86,87,88,89].

## 6. Future Perspectives

As we look to the future of molecular pharmacology, it is clear that the field will become ever more interdisciplinary and technology-driven. One exciting frontier is the integration of artificial intelligence and machine learning into drug discovery and pharmacokinetic/pharmacodynamic modeling. Computational tools are now routinely used to predict molecular properties, identify drug–target interactions, and optimize lead compounds in silico, dramatically accelerating early-stage research. For example, deep learning models trained on large chemical and bioactivity datasets can predict a compound’s bioactivity or toxicity with increasing accuracy, guiding chemists away from less promising candidates [90,91,92,93,94]. Moreover, machine learning is enhancing precision medicine by enabling the integration of multi-omics data (genomic, proteomic, metabolomic) with clinical information to forecast individual drug responses. We foresee that clinical pharmacology will increasingly incorporate algorithms that suggest optimal drugs or doses for a patient based on their molecular profile, a vision already taking shape in oncology and expected to expand to other fields. Another burgeoning area is the application of systems pharmacology and network models to understand polypharmacy and complex drug effects. By modeling the human body as an interconnected network of pathways, researchers aim to predict how modulating one target affects others—crucial for tackling multifactorial diseases like neurodegeneration, where combination therapies might be needed [95,96,97]. Fundamental research remains the engine driving these innovations, but the toolbox of molecular pharmacology is constantly growing—from CRISPR-based genome editors as potential therapeutics, to microbiome-targeting agents, to nanomedicines that deliver drugs with cellular precision [98,99,100]. Each new technology will bring its own pharmacological principles to elucidate. Encouragingly, the field has shown an admirable capacity to evolve and integrate new knowledge. The COVID-19 pandemic illustrated how quickly molecular pharmacologists and drug developers could respond, producing novel antivirals and vaccines in record time, guided by fundamental virology and immunology insights. This agility bodes well for addressing other pressing health challenges, from antibiotic resistance to rare genetic disorders [101,102].

In summary, molecular pharmacology has matured into a central pillar of modern biomedical science, linking bench-top discoveries to bedside cures. The past few years alone have seen the consolidation of precision medicine, the rise of new drug modalities, and the creative repurposing of natural scaffolds—all underpinned by deep molecular understanding. Our community continues to celebrate landmark successes, such as life-saving targeted therapies and paradigm-shifting vaccines, even as we remain vigilant about areas of uncertainty and opportunity. Looking ahead, the field is poised to advance on multiple fronts: deeper mechanistic insights through fundamental research will feed the pipeline of drug targets; improved computational and experimental tools will refine how we design and test drugs; and personalized strategies will ensure treatments are more effective and safer for each individual. The future of molecular pharmacology is therefore both promising and demanding: novel therapies for currently intractable diseases are on the horizon, but developing and deploying them will require continued interdisciplinary collaboration, ethical use of genetic data, and a commitment to translating scientific discoveries into tangible health benefits [103]. As a scientific community, we stand ready to address these challenges. By building on the solid foundation of molecular and pharmacological knowledge, and by embracing innovation while remaining rooted in rigorous experimentation, molecular pharmacology will continue to thrive and deliver on its ultimate mission: to provide ever more precise, potent, and patient-centered therapeutics for the betterment of global health.
